# 42 Egyptian evidence-based consensus recommendations for diagnosis and targeted management of juvenile dermatomyositis. An initiative by the Egyptian College of Pediatric Rheumatology

**DOI:** 10.1093/rheumatology/keac496.038

**Published:** 2022-10-07

**Authors:** Y El Miedany, H Lotfy, S Salah, M Yehia, D. M Mosa, A Kaber, M Mortada, S. A Tabra, M El Gaafary, H Abdulhady, W. A Hassan, N. S Osman, M Eissa, B. M Medhat, S. S Mohamed, Y Farag, Y Amer, S. Esam Maher, A Radwan, Amira T El-Shanawany, D El Mikkawy, G El Deriny, N. A Fouad, S. I Nasef, N. E Elkaraly, M. H Abu-Zaid, Egyptian College of Pediatric Rheumatology

**Affiliations:** 1 Canterbury Christ Church University, England; 2 Rheumatology and Rehabilitation, Ain Shams University, Egypt; 3 Pediatric Rheumatology, Cairo, University, Egypt; 4 Rheumatology and Rehabilitation, Benha University, Egypt; 5 Rheumatology and Rehabilitation, Mansura University, Egypt; 6 Rheumatology and Rehabilitation, South Valley University, Egypt; 7 Rheumatology and Rehabilitation, Zagazig University, Egypt; 8 Rheumatology and Rehabilitation, Tanta University, Egypt; 9 Community medicine and Public Health, Ain Shams University, Egypt; 10 Pediatric Rheumatology, Assuit University, Egypt; 11 Rheumatology and Rehabilitation, Cairo University, Egypt; 12 Pediatric Rheumatology, Minia University, Egypt; 13 Rheumatology and Rehabilitation, Sohag University, Egypt; 14 Rheumatology and Rehabilitation, Menoufia University, Egypt; 15 Pediatric Rheumatology, Alexandria University, Egypt; 16 Rheumatology and Rehabilitation, Fayoum University, Egypt; 17 Rheumatology and Rehabilitation, Suez Canal University, Egypt

## Abstract

**Background:**

Juvenile dermatomyositis (JDM) is the most common type of juvenile idiopathic inflammatory myopathy mainly affecting the skin and proximal muscles. To date, there are no Egyptian nationally agreed, evidence-based guidelines concerning the diagnosis and management of JDM in children and treatment is often based on clinical expertise. These facts prompted us to develop clinical practice guidance for JDM suitable for patients in Egypt. This work was an initiative by the Egyptian College of Pediatric Rheumatology aiming at optimizing the management approaches for children and young adults with JDM.

**Objective:**

To provide evidence-based consensus recommendations for diagnosis and management of JDM.

**Methods:**

This study was carried out to achieve an Egyptian expert consensus on a management strategy for JDM using Delphi technique. A multistep process strategy was adopted, which started by developing 16 key clinical questions by scientific committee according to the Patient/Population, Intervention, Comparison, Outcomes and Time (PICOT) approach. The core leadership team identified clinicians and researchers with expertise in pediatric rheumatology all over Egypt. An evidence-based, systematic, literature review was conducted to compile evidence for the JDM management. Delphi process was implemented (3-rounds) to reach a consensus.

**Results:**

Twenty-five expert panel participated in the 3 rounds with response rate 100%. A total of 22 recommendations, categorized into 15 domains (Targeted population, diagnostic criteria, high risk patients, diagnostic criteria, definition of disease activity status, differences between JDM and DM in adults, lab tests, treatment targets, treatment, monitoring, resisted cases, drug tapering, comorbidities, prognosis, vaccination and transition program. The Agreement with the recommendations (rank 7–9) ranged from 85.2–95.2%. The Consensus was reached (i.e. ≥75% of respondents strongly agreed or agreed) on all the clinical standards. Algorithm for management has also been developed.

**Conclusion:**

This work provided an updated management approach for JDM patients. This evidence-based informed consensus process is expected to support uniform, high quality standards of care for children with JDM in Egypt

**The implication to policy, practice, research and advocacy:**

to provide updated recommendations for better management of JDM.

